# Targeting Unconventional Pathways in Pursuit of Novel Antifungals

**DOI:** 10.3389/fmolb.2020.621366

**Published:** 2021-01-12

**Authors:** Stephanie Nguyen, Jia Q. Truong, John B. Bruning

**Affiliations:** ^1^Institute of Photonics and Advanced Sensing (IPAS), School of Biological Sciences, The University of Adelaide, Adelaide, SA, Australia; ^2^School of Biological Sciences, The University of Adelaide, Adelaide, SA, Australia

**Keywords:** *Aspergillus fumigatus*, *Candida albicans*, *Cryptococcus neoformans*, drug targets, drug discovery, antifungal

## Abstract

The impact of invasive fungal infections on human health is a serious, but largely overlooked, public health issue. Commonly affecting the immunocompromised community, fungal infections are predominantly caused by species of *Candida, Cryptococcus*, and *Aspergillus*. Treatments are reliant on the aggressive use of pre-existing antifungal drug classes that target the fungal cell wall and membrane. Despite their frequent use, these drugs are subject to unfavorable drug-drug interactions, can cause undesirable side-effects and have compromised efficacy due to the emergence of antifungal resistance. Hence, there is a clear need to develop novel classes of antifungal drugs. A promising approach involves exploiting the metabolic needs of fungi by targeted interruption of essential metabolic pathways. This review highlights potential antifungal targets including enolase, a component of the enolase-plasminogen complex, and enzymes from the mannitol biosynthesis and purine nucleotide biosynthesis pathways. There has been increased interest in the enzymes that comprise these particular pathways and further investigation into their merits as antifungal targets and roles in fungal survival and virulence are warranted. Disruption of these vital processes by targeting unconventional pathways with small molecules or antibodies may serve as a promising approach to discovering novel classes of antifungals.

## Introduction

Opportunistic fungal pathogens impose a significant societal and economic burden on the public health sector, particularly amongst immunocompromised patients. In the United States alone, it has been estimated that fungal infections incur hospitalization and outpatient costs that exceed $7.2 billion USD annually (Benedict et al., 2019). These costs are expected to rise in tandem with the expansion of the immunocompromised population and subsequent increase in the incidence of invasive mycoses. This predisposed population includes recipients of invasive surgery, chemotherapy, and immunosuppressive therapy, as well as sufferers of human immunodeficiency virus (HIV)/acquired immunodeficiency syndrome (AIDS) and cystic fibrosis (Nucci and Marr, 2005).

Global estimates suggest that over 150 million individuals are affected by serious fungal infections which culminate in over 1.6 million deaths per annum (Bongomin et al., 2017). Common fungal pathogens associated with these opportunistic infections include *Aspergillus* spp. (most commonly *Aspergillus fumigatus*), *Candida albicans* (and other non-*albicans Candida* species), *Cryptococcus neoformans, Histoplasma capsulatum, Coccidioides imitis*, and *Pneumocystis jirovecii* (Walsh and Dixon, 1996; Vandeputte et al., 2012). Amongst these fungal pathogens, species of *Aspergillus, Candida, Pneumocystis*, and *Cryptococcus* are responsible for over 90% of total global fungal mortalities and therefore, these fungal species pose the most prominent threat to human health (Brown et al., 2012). Aspergillosis, candidiasis, and cryptococcosis often manifest as deep tissue mycoses that occupy different niches within the mammalian host. *A. fumigatus* preferentially colonizes lung tissue, resulting in pulmonary aspergillosis whereas *C. albicans* primarily infects the blood, resulting in systemic candidiasis, and *C. neoformans* establishes an initial infection in the lungs, causing pulmonary cryptococcosis (Latgé, 1999; Wenzel and Gennings, 2005; Sabiiti and May, 2012). The severity of these diseases can escalate rapidly as the infection disseminates throughout the body and affects multiple organs. Infections caused by *A. fumigatus* and *C. albicans* can manifest as invasive aspergillosis and invasive candidiasis, respectively, both of which target the kidney and brain, whereas *C. neoformans* can cause cryptococcal meningitis which affects the central nervous system (Bicanic and Harrison, 2005; Schmiedel and Zimmerli, 2016; Shi and Mody, 2016).

In this advanced stage, treatment of invasive mycoses requires aggressive and expensive antifungal therapy. However, the success of treatment is often impeded by (i) fundamental issues in the diagnostic stage, (ii) unfavorable characteristics that are inherent to pre-existing antifungal drugs or the (iii) emergence of antifungal resistance, all of which ultimately lead to primary antifungal therapy failure (Nucci and Perfect, 2008). In patients suffering from invasive candidiasis or invasive aspergillosis, the rate of failure can be as high as 60 and 70%, respectively (Nucci and Perfect, 2008). To address these issues, there have been advancements in diagnostic techniques and several iterations of pre-existing drugs have been developed to improve their pharmacological properties (Houšt et al., 2020; Kidd et al., 2020). However, new classes of antifungal drugs that bypass existing resistance mechanisms by targeting alternate pathways are yet to be discovered.

In spite of the alarming rates of morbidity and mortality, the severity of invasive fungal infections remains underappreciated. Increases in disease incidence and prevalence of antifungal resistance highlights the need to identify novel targets and develop new classes of antifungals to manage mycoses amongst the immunocompromised population. There have been continual efforts to characterize enzymes involved in the biosynthesis of ergosterol or cell wall components, both of which are classic antifungal targets, to develop novel inhibitors (Urbina et al., 2000; Hata et al., 2010; Marshall et al., 2018). However, there has also been a notable shift in focus from these pathways exclusive to fungi to exploiting species-specific differences in shared pathways between fungi and humans (Rodriguez-Suarez et al., 2007; Marshall et al., 2017, 2019; Kummari et al., 2018). To effectively establish infection, the fungus must adapt to a different niche within the human host, combat, or circumvent the host immune response and obtain sufficient nutrients to reproduce and disseminate. Although these metabolic requirements may differ between fungal species, depending on their preferred infection site, disrupting shared metabolic pathways involved in these processes can impede fungal survival and pathogenesis. Targeting these pathways may present an elegant approach to develop novel classes of therapeutics with broad-spectrum activity.

In this article, we have focused primarily on emerging targets for the development of novel antifungal classes. We have identified key enzymes involved in several targetable metabolic pathways and consolidated extensive research to define their roles in fungal survival and virulence. Furthermore, we have discussed their merits as potential targets and provided practical discussions to drive future drug design efforts from bench to bedside.

## Current Antifungal Classes and Treatments

**Figure 1:**
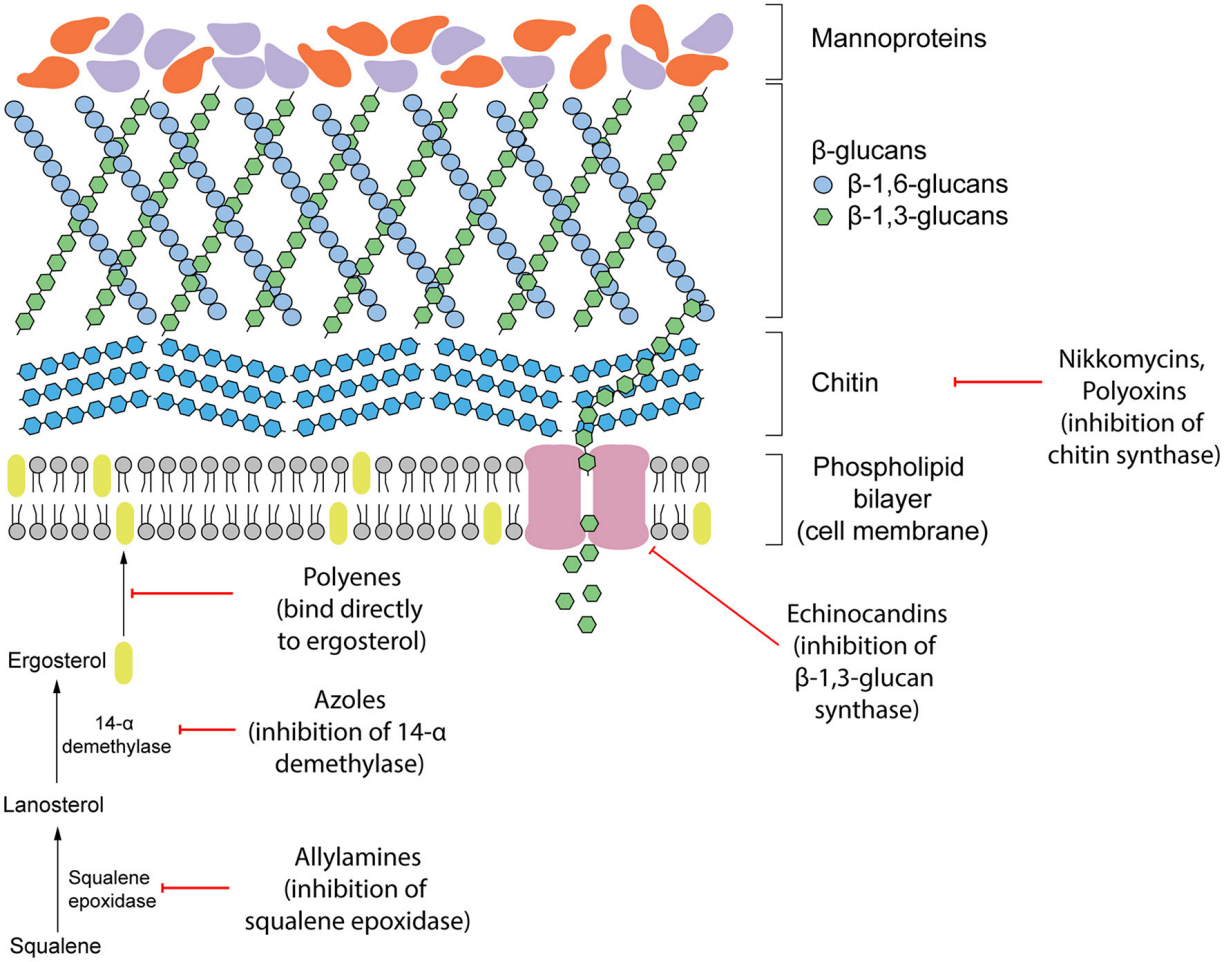
Existing antifungal drug classes that target the biosynthesis of the fungal cell wall components (nikkomycins, polyoxins, echinocandins), biosynthesis of the cell membrane (azoles, allylamines) or bind to ergosterol in the membrane directly (polyenes).

Antifungal therapeutics are routinely administered to combat invasive mycoses. The four main classes of antifungals that currently exist function by targeting either the cell wall or cell membrane (Figure 1). They include azoles, allylamines, polyenes and echinocandins. The mechanism of action of each antifungal drug class and discussion of their biological targets have been extensively reviewed (Mazu et al., 2016). In this review, we have provided a brief overview of the currently available antifungals.

### Targeting the Cell Membrane

Ergosterol is a steroid alcohol that forms a major component of the fungal cell membrane. It is responsible for the maintenance of membrane fluidity and structural integrity, as well as the regulation of membrane permeability (Gooday, 1995). Due to its implications in fungal survival, ergosterol biosynthesis enzymes have been the target of the allylamine and azole classes of antifungals, whereas the polyene class targets ergosterol function directly (Mazu et al., 2016). Allylamines target squalene epoxidase, an enzyme that catalyzes the conversion of squalene to lanosterol whereas azoles function as non-competitive inhibitors of lanosterol 14α-demethylase by complexing with the haem prosthetic group (Ryder, 1988; Allen et al., 2015; Mazu et al., 2016). Both of these enzymes are involved in the later stages of ergosterol biosynthesis. Disruption of this pathway leads to an accumulation of upstream sterol precursors and ultimately, the depletion of ergosterol. This deficiency results in impaired cell membrane function, growth inhibition, and cell death (Ghannoum and Rice, 1999; Allen et al., 2015). In comparison, polyenes induce fungal death by binding directly to ergosterol embedded in the cell membrane. Once incorporated into the membrane, this leads to the formation of pores which increase membrane permeability to water, ions and non-electrolytes (Ermishkin et al., 1976).

### Targeting the Cell Wall

Due to the absence of a mammalian equivalent, several components of the fungal cell wall and the biosynthetic enzymes responsible for its construction have been the target for antifungal therapeutics. The cell wall, comprised of three main components including glucans, mannoproteins, and chitin, plays an important role in maintaining fungal survival by maintaining cell rigidity and resisting osmotic stress (Gow et al., 2017). Furthermore, there are several receptors displayed on the surface of the cell wall that can interact with the host during infection and contribute to fungal virulence. Echinocandins disrupt these fundamental functions of the cell wall by non-competitively inhibiting (1,3)-β-glucan synthase, the enzyme responsible for maintaining and synthesizing glucans. Insufficient biosynthesis of glucans leads to destabilization of the cell wall and a compromised ability to resist osmotic pressure that results in cell lysis (Wiederhold and Lewis, 2003).

## Emergence of Antifungal Resistance and the Need for Novel Antifungal Classes

The extensive use of antifungals in both agricultural and clinical settings has led to the rise of widespread antifungal resistance with significant consequences to food security and human health. To control fungal phytopathogens in agricultural crops, a range of antifungal drugs specifically for agricultural use are used that target mitochondrial respiration processes, including succinate dehydrogenase and Qo inhibitors (Brauer et al., 2019). However, there is also significant overlap in the use of azoles to treat both human and plant fungal pathogens. In particular, they are frequently used in grain- and grass-growing environments, as well as in clinical settings, to control *Candida* spp., *Cryptococcus* spp. and *Aspergillus* spp. infections (Azevedo et al., 2015). As a result, bioactive azoles can persist in soil, water, and fresh produce (Hof, 2001). Ultimately, it has been suggested that these environmental factors contribute to the rise in azole resistance currently being observed in clinical settings.

The prominent use of azoles has resulted in a shift in the prevalence of infections caused by *C. albicans* toward less susceptible species of *Candida*, including *C. glabrata, C. krusei*, and *C. guilliermondii* (Hope et al., 2002; Pfaller et al., 2006; Perlin et al., 2015). Fungal strains can also acquire drug resistance during therapy which restricts the treatment options available to the patient and can ultimately cause antifungal therapy failure. Specifically, there are increasing concerns of echinocandin and azole resistance arising in species of *Candida* and *A. fumigatus*, respectively (Perlin, 2015). In the UK, there has been a consistent increase in the proportion of *A. fumigatus* isolates with resistance to azoles growing from 5% in 2004 to 14% in 2008 and 20% in 2009 (Bueid et al., 2010). Following a similar trend, the frequency of *A. fumigatus* clinical isolates with azole resistance in The Netherlands increased from 7.6% in 2013 to 14.7% in 2018 (Lestrade et al., 2020).

Several generations of antifungal drugs originating from these four classes have been developed to improve their pharmacokinetic profiles, increase potency, and combat resistance. However, treatment of invasive mycoses amongst immunocompromised patients remains challenging. Primary antifungal therapy failure is a prevalent issue, affecting 20–60% of invasive candidiasis patients, 40–60% of invasive aspergillosis patients and 30–100% of invasive fusariosis patients (Nucci and Perfect, 2008). Although the role of underlying host factors and immune status must be considered in antifungal therapy failure, several issues arise from inherent limitations in currently available drugs. Low bioavailability in target tissues, drug toxicity, unfavorable drug-drug interactions, and the emergence of drug resistance also contribute to the reduced efficacy of existing treatments (Nucci and Perfect, 2008).

As the number of antifungal classes available for clinical use is limited and the proportion of the population susceptible to invasive mycoses is expected to increase, there is a need to identify new targets for the design and development of novel classes of antifungals. Although cell membrane and cell wall biosynthesis enzymes have been traditional targets of antifungals, broadening our search to unconventional pathways can unlock an abundance of potential targets by exploiting the unique metabolic needs of fungi during pathogenesis. A summary of the proposed targets discussed in this review is outlined in Table 1.

**Table 1:** Cellular function of the proposed antifungal protein targets and phenotypic characteristics of various fungi after genetic knockout/knockdown.

	**Protein targets for antifungal drugs**						
	**Enolase**	**Mannitol-2-Dehydrogenase**	**mannitol-1-phosphate 5-dehydrogenase**	**Inosine monophosphate dehydrogenase**	**Guanosine monophosphate synthase**	**Nucleoside diphosphate kinase**	**References**
Roles in fungi	• Catalyzes the penultimate step of glycolysis • Expressed on the surface and functions as a receptor for human plasminogen • Contributes to tissue invasion and nutrient acquisition during infection	• Biosynthesis of mannitol, a storage of carbohydrates, an osmolyte and source of reducing power • Quenches reactive oxygen species • Protects conidia against stressful conditions		• Biosynthesis of purine nucleobases required for signal transduction pathways, energy metabolism and DNA and RNA synthesis			Meena et al., 2015; Funk et al., 2016; Ji et al., 2016; Chitty and Fraser, 2017
**Phenotype of genetic knockout/knockdown**							
*Aspergillus fumigatus*					• Growth defects that could be recovered with exogenous guanine • Avirulent in a murine model of infection	• Essential for fungal survival	Rodriguez-Suarez et al., 2007; Dinamarco et al., 2012
*Candida albicans*	• Reduced germination tube and hyphal formation • Attenuated virulence and growth rate				• Growth defects that could be recovered with exogenous guanine • Avirulent in a murine model of infection		De Backer et al., 2001; Rodriguez-Suarez et al., 2007; Ko et al., 2013
*Cryptococcus neoformans*		• Decreased tolerance to high temperatures and salinity^*^ • Decreased resistance to oxidative stress • 5,000-fold less virulent compared to wild-type *C. neoformans* H99 in a mouse model		• Reduced growth rates • Impaired expression of virulence factors ◦ Smaller capsule size ◦ Reduced melanin expression • Avirulent in a murine model of cryptococcus	• Growth defects that could be recovered with exogenous guanine • Avirulent in a murine model of infection • Impaired expression of virulence factors◦ Smaller capsule ◦ Delayed melanin production ◦ No detectable protease activity		Chaturvedi et al., 1996a,b; Morrow et al., 2012; Chitty et al., 2017
*Aspergillus niger*			• Decreased tolerance to high temperatures and oxidative stress • Greater sensitivity to freeze-drying and freeze-thawing				Ruijter et al., 2003
*Aspergillus fischeri*			• Modest increase in sensitivity to heat and oxidative stress in conidia				Wyatt et al., 2014
*Aspergillus flavus*						• Disruption of one of two copies impairs conidia and sclerotia development • Affects plant virulence in a maize and peanut seed model	Wang et al., 2019
*Aspergillus nidulans*						• Essential for fungal survival • Essential for hyphal growth and conidia production	Lin et al., 2003

* *These observations are based on the phenotype of a mutant Cryptococcus neoformans strain that produces low levels of mannitol. However, the mutation that causes this phenotype has not been determined*.

## Emerging Targets for New Antifungal Development

### Glycolysis Pathway

Glycolysis is a fundamental, multi-step metabolic process that occurs in most living organisms. The glycolysis pathway produces energy, in aerobic and anaerobic conditions, through the catabolism of sugars and provides useful intermediates for downstream biosynthetic pathways. The roles of these enzymes in glycolysis have been extensively studied but there has been growing interest in their additional “moonlighting” functions that deviate from their canonical function. These “moonlighting” functions may contribute to fungal survival and virulence mechanisms and have consequently been investigated as promising antifungal drug targets.

### Enolase and Host Plasminogen

**Figure 2:**
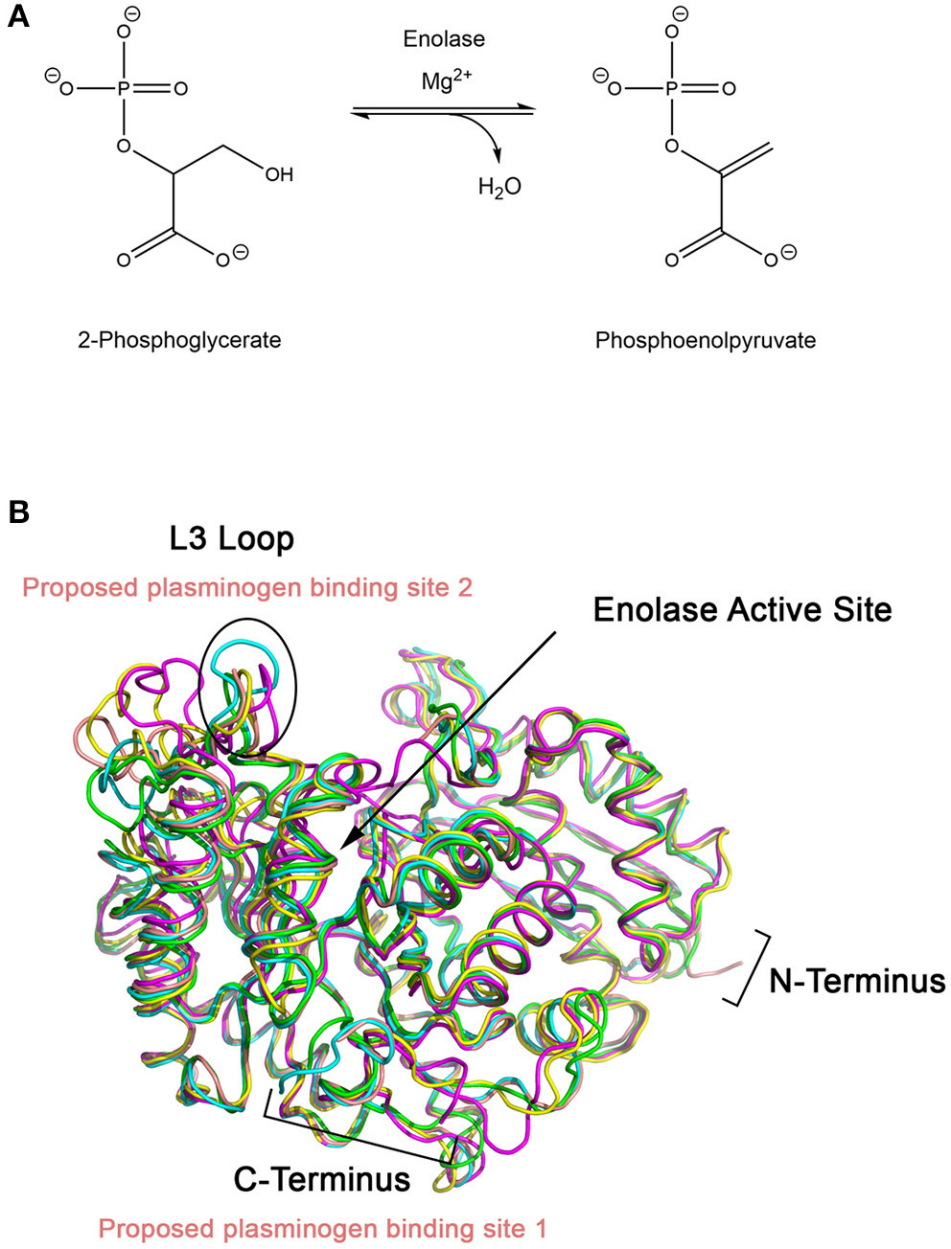
**(A)** Enolase catalyzes the penultimate step of glycolysis, interconverting 2-phosphoglycerate (2-PG) and phosphoenolpyruvate (PEP) using magnesium as a cofactor. **(B)** X-ray crystal structures of enolase from different species have similar global folds. Enolase structures from *Escherichia coli* (1E9I; green), *Enterococcus hirae* (1IYX; cyan), lobster (1PDZ; yellow), *Streptococcus pneumoniae* (1W6T; salmon), and *Saccharomyces cerevisiae* (1ONE; magenta) were superimposed using *PyMOL* (Stec and Lebioda, 1990; Duquerroy et al., 1995; Kuhnel and Luisi, 2001; Hosaka et al., 2003; Ehinger et al., 2004; Kang et al., 2008). The L3 loop, which shows the greatest structural deviation between the structures, is proposed to be the plasminogen binding loop for *S. pneumoniae*. Exposed lysine residues at the C-terminal end of enolase have also been proposed as a possible binding site.

Enolase catalyzes the penultimate step of glycolysis, interconverting 2-phosphoglycerate (2-PG) and phosphoenolpyruvate (PEP) (Figure 2) (Ji et al., 2016). Consistent with its role in integral metabolic processes, a genetic knockout of enolase in *C. albicans* has shown to reduce germination tube and hyphal formation, resulting in attenuated virulence and growth rate (De Backer et al., 2001; Ko et al., 2013). Intriguingly, this enzyme fulfills its glycolytic roles in the cytoplasm but it is also expressed on the cell surface of many fungal and bacterial species. This has been observed in *A. fumigatus* and *C. albicans*, two of the most prominent etiological agents of invasive mycoses, as well as *Aspergillus flavus, Aspergillus terrus, Aspergillus nidulans, Candida glabrata, Saccharomyces cerevisiae* and bacterial *Streptococci* and *Pneumococci* species (Pancholi and Fischetti, 1998; Edwards et al., 1999; Bergmann et al., 2001; Funk et al., 2016). In *S. cerevisiae*, it has been suggested that enolase secretion is mediated by an N-terminal 28 amino acid translocation sequence and operates through a SNARE-dependent pathway (Miura et al., 2012). Alignment of homologous enolase protein sequences from *S. cerevisiae* with *Aspergillus* spp. and *Candida* spp. have identified a highly conserved motif in the N-terminus. This suggests that this SNARE-dependent mechanism of enolase secretion may be conserved amongst many fungal species (Funk et al., 2016).

The function of surface-expressed enolase deviates from its canonical role in the cytoplasm and instead contributes to tissue invasion and nutrient acquisition during pathogenesis. It functions as a receptor for plasminogen, a zymogen circulating in the host bloodstream. Plasminogen is cleaved by plasminogen activating proteins (including tissue-plasminogen activator and urokinase plasminogen activator) to form active plasmin which functions as a serine protease. The physiological function of the human plasminogen/plasmin system is to facilitate tissue remodeling, cell migration, hemostasis and wound healing, and induce inflammation and angiogenesis. It fulfills these roles by degrading proteins in the extracellular matrix, fibrin and fibrinogen in blood clots and targeting components of the complement system (Law et al., 2013). Inactive plasminogen is proposed to dock onto a lysine-rich motif located on the surface-expressed enolase of fungal pathogens. While bound, host plasminogen activators are capable of recognizing, binding, and cleaving plasminogen to produce the activated plasmin (Funk et al., 2016). The serine protease activity of plasmin becomes concentrated at the site of fungal infection and this can accelerate tissue invasion and disease progression. An in-depth study which focused on surface-expressed enolase from *A. fumigatus* confirmed its ability to interact with human immune regulators including factor H, factor-H-like protein 1 (FHL-1), C4b-binding protein (C4BP) and plasminogen (Dasari et al., 2019). Whilst bound to *A. fumigatus* enolase, factor H, FHL-1 and C4BP retained their normal cellular activity and plasminogen remained accessible to plasminogen activator proteins. When swollen *A. fumigatus* conidia coated with human plasminogen were exposed to human A549 epithelial cells or an epithelial monolayer, cellular metabolic activity was reduced by 41% and cell retraction was reduced (Dasari et al., 2019). These observations are consistent with the “moonlighting” role of surface-expressed enolase and their role in facilitating tissue invasion.

### Immunogenic Activity of Enolase

The secretion and presentation of enolase on the cell surface is a common feature of many pathogenic fungal species (Edwards et al., 1999; Funk et al., 2016). As a surface-exposed receptor, it is capable of eliciting an immunogenic response that may be exploited for vaccine development. Preliminary studies have demonstrated the suitability of using enolase as an immunogenic agent to acquire modest protective effects against candidiasis in a murine model. When mice were challenged with *C. albicans* following subcutaneous vaccination with recombinant *C. albicans* enolase, an elevated antibody response was induced and fungal burden in major organs was reduced compared to non-immunized mice (Li et al., 2011). In an independent study, *C. albicans* enolase was expressed and presented on *Lactobacillus casei* cells, then orally administered to mice prior to challenging with a normally lethal dose of *C. albicans*. These immunized mice produced higher IgG antibody titers and had improved survival rates (Shibasaki et al., 2014).

### Targeting Surface-Expressed Enolase

**Figure 3:**
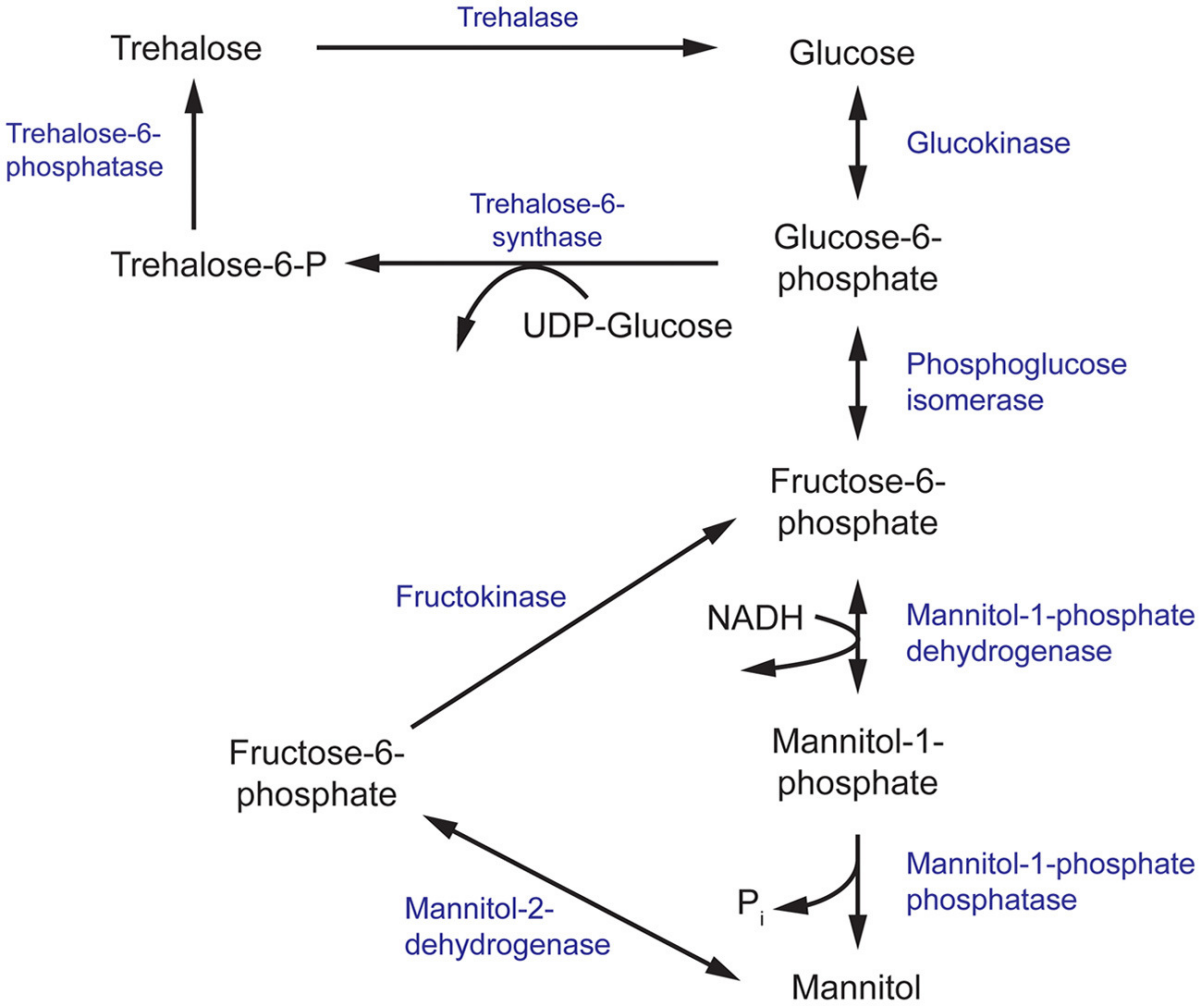
The mannitol and trehalose biosynthesis pathway in fungi. The primary biosynthesis enzymes of mannitol, and the focus of this review, are mannitol-1-phosphate dehydrogenase (M1P5DH) and mannitol-2-dehydrogenase (M2D).

Designing a peptidomimetic or small molecule inhibitor that specifically targets the plasminogen docking site of enolase using a structure-guided approach is a possible avenue for new therapeutics. This approach requires intimate knowledge of the interactions between both proteins. Although high resolution crystal structures of the full-length human Type II plasminogen (4DUR) and fungal enolase from *S. cerevisiae* (3ENL) are already available, the interaction interface has not yet been defined (Stec and Lebioda, 1990; Law et al., 2012). There have been previous studies examining the molecular interaction between plasminogen and bacterial enolases. Studies conducted using enolase from *S. pneumonia* have suggested two possible binding sites—the L3 loop on the alpha-beta (αβ) barrel, a structurally flexible region, or the lysine-rich C-terminal tail of the protein (Pancholi and Fischetti, 1998; Bergmann et al., 2003; Ehinger et al., 2004). Superimposition of enolase from prokaryotic and eukaryotic sources including *E. coli* (1E9I), human (3B97), *Enterococcus hirae* (1IYX), lobster (1PDZ), *Streptococcus pneumonia* (1W6T), and *S. cerevisiae* (3ENL) reveal an overall similar fold and existence of a conserved, but structurally different, L3 loop (Figure 3) (Stec and Lebioda, 1990; Duquerroy et al., 1995; Kuhnel and Luisi, 2001; Hosaka et al., 2003; Ehinger et al., 2004; Kang et al., 2008). Therefore, it is likely that the plasminogen binding site is also conserved between homologous enolase enzymes but the mode of binding may differ between species. Building upon this work, efforts must be focused on elucidating the plasminogen binding interface of fungal enolase and structurally characterizing this site to guide rational inhibitor design.

Evidence of the immunogenicity of fungal enolase, particularly the *C. albicans* homolog, raises the possibility of using antibody therapy as an adjunct to antifungal therapy. Therapeutic antibodies specific for enolase could potentially reduce tissue invasion and fungal dissemination by interfering with interactions between fungal enolase and host plasminogen. Antibodies raised in mice against recombinant *C. albicans* or *A. fumigatus* enolase successfully recognized plasminogen-bound enolase from *Candida* spp. and *Aspergillus* spp., respectively. However, this antibody-enolase-plasminogen complex still allowed plasminogen activating proteins to access, cleave, and activate bound plasminogen (Funk et al., 2016). The viability of antibody therapy requires improved antibodies against enolase that recognize specific epitopes that interfere with this plasminogen activation process. Monoclonal antibodies that inhibit plasminogen activation have already been successfully raised against human α-enolase. During the process of fibrinolysis, plasminogen binds to surface-expressed α-enolase on leukocytic cells and this results in an accelerated rate of plasminogen activation. The plasminogen activation associated with binding to leukocytic cell lines was reduced by ~90% when monoclonal α-enolase antibodies (MAb 11G1) were introduced (López-Alemany et al., 2003). These experiments provide a proof-of-principle that disruption of an enolase-plasminogen interaction can affect plasmin activity and therefore, this approach may be feasible when applied to an antifungal therapeutic strategy.

## Mannitol Biosynthesis Pathway

Mannitol biosynthesis is a crucial process that aids in fungal survivability and virulence. Mannitol-2-dehydrogenase (M2DH) and mannitol-1-phosphate 5-dehydrogenase (M1P5DH) are the primary biosynthesis enzymes that produce mannitol, an acyclic, six-carbon sterol alcohol (Figure 3). Present in all fungal structures, including mycelia, fruiting bodies and conidia, mannitol acts as a carbohydrate storage molecule, osmolyte, and source of reducing power (Meena et al., 2015). During pathogenesis, mannitol production is increased to exploit its ability to quench reactive oxygen species (ROS) in order to resist host defenses (Meena et al., 2015). The unique and diverse properties of mannitol, particularly in relation to human pathogenesis, have garnered considerable interest in the underlying mechanism of its biosynthesis and secretion during fungal infection.

### Mannitol as a Biomarker of Fungal Infection

The efficacy of antifungal therapy is dependent on the early detection and accurate identification of the invading fungal species. Traditional methods include culturing from patient samples, direct microscopical examination of fungal samples and histopathology testing of infected tissue (Kozel and Wickes, 2014). Although culturing is considered as the gold standard of diagnosis, the utility of this technique can suffer from the slow growth rates of filamentous fungi, environmental contamination, and poor recovery of some fungal species from patient samples (Kozel and Wickes, 2014). Overall, these factors can delay adequate antifungal treatment which can compromise the efficacy of these drugs. Therefore, non-culture-based methods of diagnosis, such as screening patient samples for biomarkers of fungal infection, offer a faster and less invasive alternative.

Several fungal pathogens, including *C. neoformans* and *Aspergillus* spp., have been shown to produce high levels of mannitol *in vitro* and in animal disease models of infection, highlighting the suitability for its use as a biomarker. In liquid cultures of 12 human isolates of *C. neoformans*, extracellular mannitol levels increased ~30-fold over a 4 day period in which rapid mannitol biosynthesis and secretion occurred predominantly in the stationary growth phase (Wong et al., 1990). Similarly, several *Aspergillus* species have also demonstrated an ability to produce mannitol from glucose *in vitro* (Birkinshaw et al., 1931). Closely mirroring these studies, mannitol levels in the cerebrospinal fluid (CSF) of a cortisone-treated rabbit disease model of cryptococcal meningitis also increased over time in response to infection with *C. neoformans* strain H99. Furthermore, there was a positive correlation identified between mannitol CSF levels and CSF colony forming units as well as CSF cryptococcal antigen titers, both of which are markers of infection severity (Wong et al., 1990). In an experimental model of aspergillosis using rats infected with *A. fumigatus*, high levels of mannitol were detected in liver tissue 12 h post-infection and in serum 36 h post-infection (Wong et al., 1989). In addition, a singularly surviving rat 48 h post-infection also showed increased mannitol levels in lung, liver, and kidney tissue (Wong et al., 1989). From these animal models, the results indicate that infection of a human host with *C. neoformans* or *A. fumigatus* is also likely to induce upregulated mannitol production and secretion, localizing in the CSF or serum, respectively. Analysis of the CSF from patients suffering from AIDS and cryptococcal meningitis confirm that mannitol content increases to sufficiently high levels to be easily detected and quantified using gas-liquid chromatography-linked mass spectroscopy (Megson et al., 1996). As humans lack an equivalent mannitol biosynthesis pathway, the detected mannitol is likely of fungal origin. However, mannitol content in the CSF could not be significantly correlated to cryptococcal antigen titers in humans, contrary to observations made in the rabbit disease model. Taken together, these results provide valuable insights into the feasibility of using patient CSF and serum mannitol levels as a biomarker of fungal infection and presents possible methodologies that facilitate its use as a diagnostic tool.

### Roles in Virulence and Protection Against Host Defenses

Fungal pathogens invading a human host experience multiple environmental stresses associated with occupying a new niche and chemical stresses invoked by the immune response. Sensing and adapting to abiotic stresses (temperature, pH), resisting phagocytosis from macrophages and neutralizing ROS produced by neutrophils is critical for survival (Cooney and Klein, 2008; Khanna et al., 2016). Mannitol has been connected to many of these host-pathogen interactions associated with fungal survival and virulence and this provides a basis to understand the function of its rapid biosynthesis and secretion during infection.

Phenotypic characterization of a *C. neoformans* mutant which produces reduced levels of mannitol (Cn MLP) exemplifies the multitude of roles adopted by mannitol. The Cn MLP mutant showed similar growth rates, morphology, and antifungal susceptibility as the wild-type strain, *C. neoformans* H99. However, Cn MLP had decreased tolerance to high temperatures and salinity (Chaturvedi et al., 1996a). These findings are consistent with the proposed role of mannitol as an osmolyte in fungi (Meena et al., 2015). Although the loss of thermotolerance in Cn MLP cannot be directly linked to mannitol function, resistance to thermal stress in the fungus *Beauveria bassiana* has been partially attributed to trehalose, a metabolite produced from mannitol (Figure 3) (Liu et al., 2008). The Cn MLP isolate also demonstrated decreased resistance to oxidative stress, a common killing mechanism of the human immune system (Chaturvedi et al., 1996b). Specifically, Cn MLP showed greater susceptibility to oxidative killing by polymorphonuclear neutrophils (PMNs) and ROS generated from an iron-hydrogen peroxide-iodide cytotoxic cell-free system, relative to the wild-type strain (Chaturvedi et al., 1996b). Protection against ROS improved in a dose-dependent manner when PMNs were incubated with mannitol. Similarly, fungicidal activity was completely abolished when mannitol was added simultaneously to the ROS cell free system (Chaturvedi et al., 1996b). In both scenarios, mannitol acted as an effective scavenger of ROS to confer protection against oxidative damage. In an experimental disease model, Cn MLP was 5,000-fold less virulent compared to *C. neoformans* H99 when intravenously injected into mice. Although both the mutant and wild-type strains proved to be lethal, 100% of mice succumbed to infection 51 days post-injection with *C. neoformans* H99 whereas 100% of mice injected with Cn MLP survived at the same time point (Chaturvedi et al., 1996a). Together, the osmotolerance and thermotolerance conferred by intracellular mannitol and the protection against ROS by extracellular mannitol may aid fungal survival and virulence by resisting environmental and immune-induced stresses in the human body.

### Roles in Conidial Stress Response

Conidia are asexually-produced spores that are required for wide-spread fungal dispersal. Often dissipated through the air, conidia are inhaled into the respiratory system and at this developmental stage, fungal infection can be initiated (Dagenais and Keller, 2009). Conidia must be able to survive and adapt to this new environment to progress to subsequent germination stages and develop into a vegetative state. Mannitol has been implicated in the protection of conidia against these stress conditions in multiple species of fungi (Chaturvedi et al., 1996a; Ruijter et al., 2003; Wyatt et al., 2014).

High concentrations of intracellular mannitol have been detected in conidia from *Aspergillus oryzae, Aspergillus clavatus* and *Aspergillus niger* (Horikoshi et al., 1965; Corina and Munday, 1971; Witteveen and Visser, 1995). In order to assess mannitol function, a M1P5DH-null mutant of *A. niger* was generated. Disruption of M1P5DH resulted in a complete absence of mannitol in mycelia and a 30% reduction of intracellular mannitol content in conidia. Prolonged exposure to conditions that had no effect on the viability of wild-type conidia, such as high temperatures and oxidative stress induced by ROS, reduced the viability of M1P5DH-null conidia to 5 and 0.5%, respectively. To a lesser extent, these mutant conidia were more sensitive to freeze-drying and freeze-thawing processes. Supplementation of M1P5DH-null conidia with mannitol had a protective effect that reduced their sensitivity to heat and oxidative stress (Ruijter et al., 2003). These results indicate that mannitol is an important molecule that protects conidia against harsh environmental conditions.

In a separate study, the equivalent gene encoding M1P5DH in *Aspergillus fischeri* was deleted with unexpected effects. There was a severe reduction of mannitol in mycelia but an increase in mannitol and reduction of trehalose in its conidia (Wyatt et al., 2014). These conidia showed a modest increase in sensitivity to heat and oxidative stress, despite the higher mannitol content. Since trehalose and mannitol biosynthesis pathways are intrinsically linked and both sugars have been found in all stages of the fungal life cycle, it has been suggested that trehalose and mannitol may play overlapping roles in resistance to environmental stress (Figure 3) (Wyatt et al., 2014; Thammahong et al., 2017). There has also been growing interest in targeting components of the trehalose biosynthesis pathway for the development of novel antifungals (Thammahong et al., 2017). Thus, further investigation into the interplay between mannitol and trehalose in fungal survival is warranted to aid these efforts.

### Targeting Mannitol Biosynthesis Enzymes

Several lines of evidence demonstrate the multifaceted roles of mannitol in the protection of fungi from stress conditions commonly experienced during infection of a host (Chaturvedi et al., 1996a; Ruijter et al., 2003; Wyatt et al., 2014). Accelerated mannitol production and secretion can confer thermotolerance and resistance to ROS generated from the human immune system needed to thrive in these harsh environments. Adding to the allure of targeting mannitol biosynthesis pathways in fungi is the fact that humans do not produce mannitol and therefore lack equivalent enzymes. This absence of a human homolog greatly simplifies the design and development of inhibitory compounds that specifically target these fungal enzymes. In turn, this will likely reduce the severity of off-target effects.

Efforts to target the mannitol biosynthesis enzyme should be focused on M1P5DH. In several species, knockout of the gene encoding M1P5DH (*mpdA*) and phenotypic characterization of these mutants have shown significant decreases in intracellular mannitol levels (Ruijter et al., 2003; Wyatt et al., 2014). Although mannitol can be synthesized in an alternate pathway using M2DH, it appears that M1P5DH features in the predominant pathway. Hence, inhibition of M1P5DH is likely to have a greater effect on conidia viability and can be useful to halt initial stages of infection and dissemination.

Targeting the mannitol biosynthesis pathway can also be beneficial to delay the progression of existing infections. As demonstrated in the animal models infected with Cn MLP, decreased mannitol levels were associated with reduced virulence and ultimately, greater survival. By inhibiting the activity of these biosynthesis enzymes, mannitol production is reduced and this may increase fungal sensitivity to oxidative killing by components of the immune system.

## Purine Nucleotide Biosynthesis Pathway

**Figure 4:**
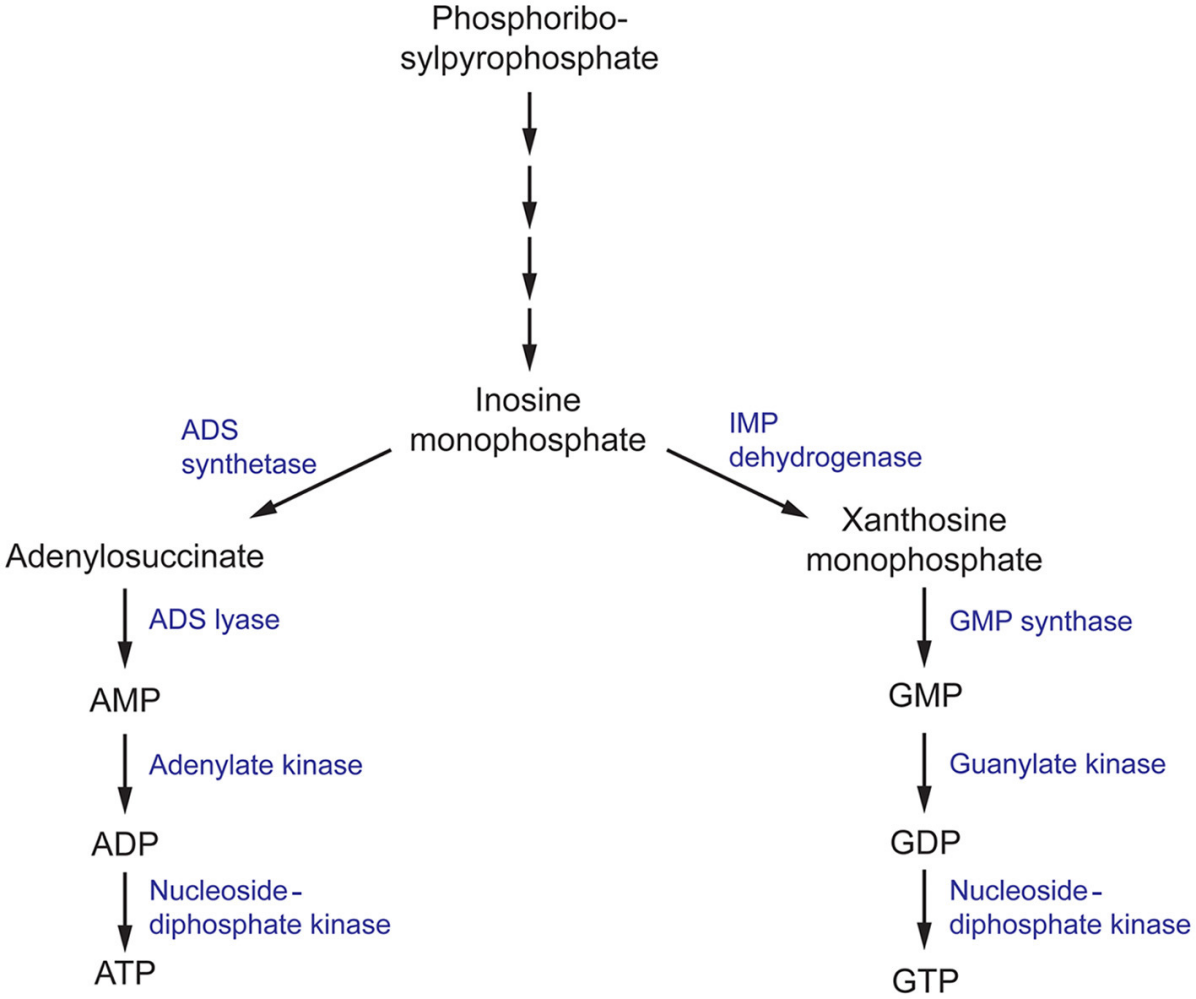
The purine metabolism pathway in fungi. ADS, (adenylosuccinate) synthetase; ADS, (adenylosuccinate) lyase; IMP (inosine monophosphate) dehydrogenase; GMP, (guanosine monophosphate) synthase.

Nucleobases, including purines and pyrimidines, are heterocyclic molecules that are found in all forms of life. They are obtained exogenously from the environment or can be synthesized endogenously. As they are involved in essential processes, including DNA and RNA synthesis, energy metabolism and signal transduction, the intracellular pool of nucleobases must be tightly maintained (Chitty and Fraser, 2017). Hence, disruption of the purine and pyrimidine biosynthesis pathway using small molecules has been explored as a potential approach to develop antimicrobials (Rodriguez-Suarez et al., 2007; Shu and Nair, 2008; Du Pré et al., 2018; Park et al., 2018; Trapero et al., 2018). *De novo* purine biosynthesis begins with phosphoribosylpyrophosphate (PRPP) which is ultimately converted to inosine monophosphate (IMP), a precursor to both adenine and guanine nucleobases, through a series of enzymatic reactions (Figure 4) (Chitty and Fraser, 2017). This section will focus on targeting enzymes responsible for the synthesis of guanine nucleobases.

### Roles in Virulence

Inosine monophosphate dehydrogenase (IMPDH) is the first committed and rate-limiting step in the guanosine triphosphate (GTP) biosynthesis pathway. Using NAD^+^ as a cofactor, it catalyzes the conversion of IMP to xanthosine monophosphate (XMP) *via* a dehydrogenase and hydrolysis reaction (Hedstrom, 2009). IMPDH catalytic activity has been closely associated with microbial virulence, and has been the target of several drug discovery projects for antibiotics, antifungals, antivirals, and anticancer therapeutics with great success (Markland et al., 2000; Floryk and Thompson, 2010; Kummari et al., 2018; Trapero et al., 2018). In terms of antifungals, there has been substantial progress in elucidating the mechanisms in which IMPDH contributes to *C. neoformans* survival and virulence. To first validate the rationality of IMPDH as an antifungal target, an IMPDH-deficient mutant of *C. neoformans* was produced and phenotypically characterized. This mutant exhibited reduced growth rates and impaired expression of virulence factors, resulting in smaller capsule sizes and reduced melanin expression (Morrow et al., 2012). Importantly, these phenotypic defects could not be completely recovered with supplementation of exogenous guanine that may have been sourced from the human host during infection. Due to the direct involvement of IMPDH in virulence factor production, IMDPH-deficient *C. neoformans* was completely avirulent in a murine model of cryptoccoccus. As a result, fungal loads in the brain were cleared 3 days post-infection, total fungal clearance was achieved 2 weeks post-infection and ultimately, this resulted in a 100% survival rate 50 days post-infection (Morrow et al., 2012). These experiments confirm that IMPDH is involved in essential processes associated with *C. neoformans* virulence and this presents a promising avenue for the design of antifungal compounds.

In the following step of the purine biosynthesis pathway, XMP produced by IMPDH is converted to guanosine monophosphate (GMP) by guanosine monophosphate synthase (GMP synthase). In a similar manner to IMPDH, GMP synthase has been extensively studied in *A. fumigatus, C. albicans*, and *C. neoformans* and has been determined to be crucial in the survival and virulence of these fungal pathogens (Rodriguez-Suarez et al., 2007; Chitty et al., 2017). A conditional knock-out of the gene encoding GMP synthase (*GUA1*) in *A. fumigatus* and *C. albicans*, and deletion in *C. neoformans*, inhibited growth on minimal media but was recovered when supplemented with exogenous guanine. These growth defects are consistent with the essential role of purines as components of DNA and RNA. Similar to the *in vivo* experiments conducted with IMPDH-deficient *C. neoformans*, disruption of *GUA1* in *A. fumigatus, C. albicans*, and *C. neoformans* also rendered these strains avirulent in a murine model of infection (Rodriguez-Suarez et al., 2007; Chitty et al., 2017). Phenotypic characterization of Δ*GUA1 C. neoformans* revealed significant differences in the production of key virulence factors that may account for the abolished virulence. Despite supplementation with exogenous guanine, Δ*GUA1 C. neoformans* mutants produced less capsule, showed delayed melanin production and lacked detectable protease activity relative to their wild-type counterparts. Collectively, these virulence factors function to aid survival by resisting host defenses and fungal dissemination. Specifically, the capsule protects against phagocytosis, melanin resists oxidative damage and secretion of proteases facilitates host tissue degradation for nutrient acquisition. Intriguingly, this effect was more pronounced at the optimum human body temperature of 37°C (Chitty et al., 2017). Since *GUA1* is a determinant of virulence in several of these clinically-prominent fungal pathogens, inhibition of GMP synthase may be an ideal approach to develop antifungal drugs with broad-spectrum activity.

### Roles in Survival

In the final steps of the purine biosynthesis pathway, GMP must be converted to an accessible form for its use in DNA and RNA synthesis and signal transduction. GMP is first converted to guanosine diphosphate and then nucleoside-diphosphate kinase (NDK) covalently attaches a phosphate moiety to form GTP (Chitty and Fraser, 2017). NDK is a promiscuous enzyme that can use all nucleoside diphosphates as substrates, with varying levels of selectivity, to generate their respective nucleoside triphosphates (Nguyen et al., in press). Hence it is also involved in the adenine nucleoside synthesis pathway to produce adenosine triphosphate (ATP) as well as the synthesis of the remaining building blocks of DNA and RNA. Consistent with these fundamental processes, in *A. nidulans*, NDK is involved in various aspects of the life cycle, including hyphal growth and conidia production which is necessary for its growth and dispersal (Lin et al., 2003). Similarly in *A. flavus*, disruption of one of the two copies of *ndk* impairs the development of conidia and sclerotia and affects plant virulence in a maize and peanut seed model (Wang et al., 2019). Identified as an essential gene for survival in *A. fumigatus* and *A. nidulans*, NDK has been identified as a promising target for antifungals against *Aspergillus* spp. (Lin et al., 2003; Dinamarco et al., 2012).

### Targeting Purine Biosynthesis Pathways

Achieving species-selective targeting of *de novo* purine biosynthesis enzymes is an important consideration in the drug design process. This is especially important since bacteria, fungi and humans share commonality in many of these enzymes (Sperling, 1988; Kilstrup et al., 2005; Chitty and Fraser, 2017). Thus, intimate structural and kinetic knowledge of the fungal and human homologs of these enzymes will be invaluable in the design of inhibitors with high potency and selectivity. Currently, structures of human IMPDH, isoforms I (Risal et al. unpublished) and II (Colby et al., 1999), GMP synthase (Welin et al., 2013), and NDK (Chen et al., 2003) have been solved as well as structures of fungal IMPDH (*C. neoformans*) (Morrow et al., 2012) and NDK (*A. fumigatus* and *A. flavus*) (Wang and Wang, 2019; Nguyen et al., in press). Availability of these structures allows identification of key active site residues that differ between fungal and human enzymes and this may be integral in the design of highly selective inhibitors.

**Figure 5:**
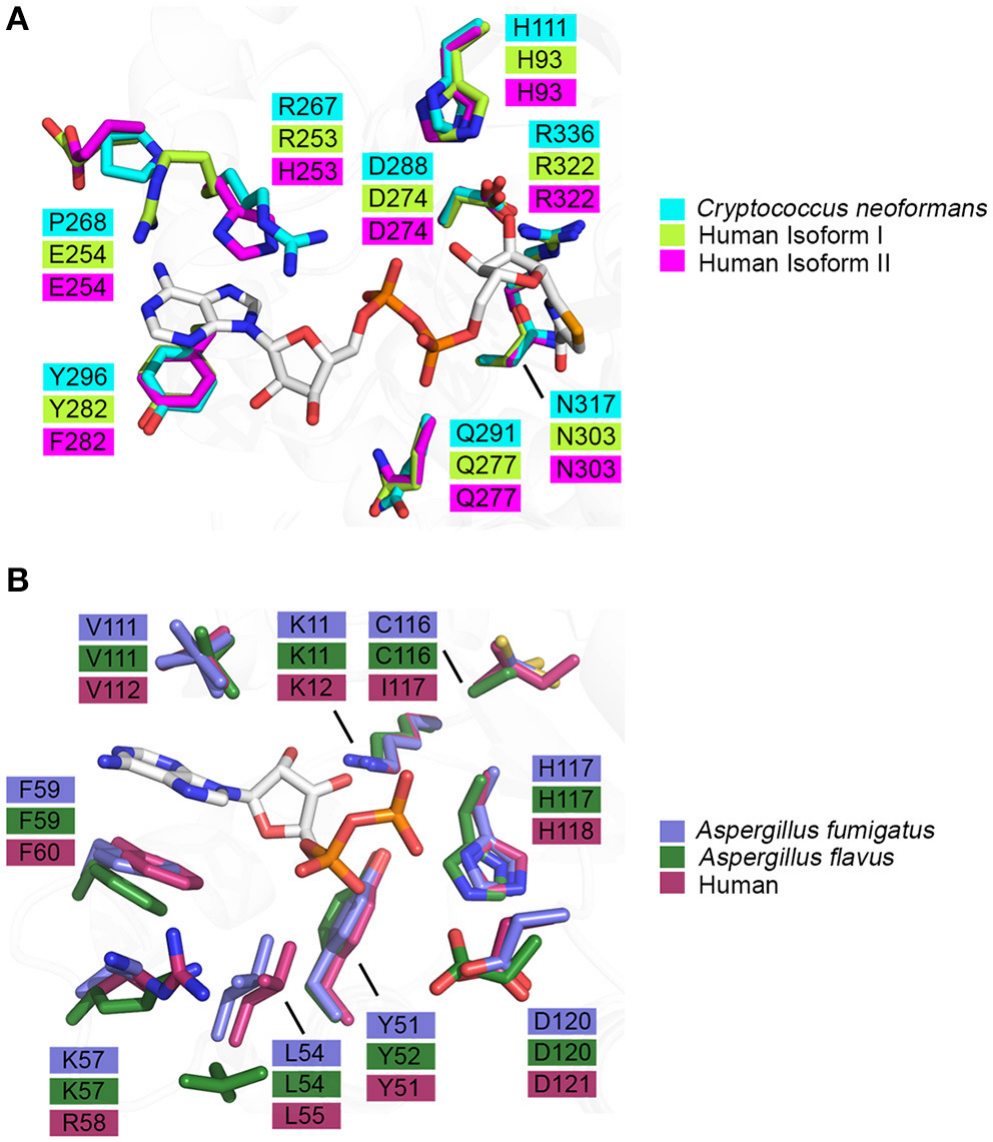
Binding pocket composition analysis reveals divergent residues between human and fungal homologs that may be exploited to achieve specificity. **(A)** Residues comprising the binding pocket of inosine monophosphate dehydrogenase from *Cryptococcus neoformans* (4AF0, cyan), human isoform I (1JCN, green), human isoform II (1B3O, magenta) and selenazole-4-carboxyamide-adenine dinucleotide, an NAD^+^ analog, are represented as sticks (Colby et al., 1999; Morrow et al., 2012) (Risal et al. unpublished). **(B)** Residues comprising the binding pocket of nucleoside di-phosphate kinase from *Aspergillus fumigatus* (6XP7, purple), *Aspergillus flavus* (6K3H, dark green) and human (2HVD, maroon) and adenosine diphosphate, a natural substrate, are represented as sticks (Giraud et al., 2006; Wang et al., 2019; Nguyen et al., in press).

Knowledge of the enzyme structure and catalytic mechanism aids in identifying the most “druggable” binding pocket. In the case of IMPDH, there are three possible sites: (i) the natural substrate site, IMP, (ii) the co-factor site, NAD^+^, or (iii) an allosteric site (Shu and Nair, 2008). Ongoing drug discovery projects targeting IMPDH from *Mycobacterium tuberculosis* and *Cryptosporidium parvum* have shown success in focusing on the co-factor binding site due to its divergence from the mammalian equivalent (Umejiego et al., 2008; Trapero et al., 2018). In the case of *C. parvum* IMPDH, a high-throughput screening approach was employed to specifically target the NAD^+^ site. Ten compounds, from a pool of 134 preliminary hits, were determined to be reversible inhibitors that were 9- to 400-fold more selective for the parasitic IMPDH over the human IMPDH isoform II (Umejiego et al., 2008). These experiments provide a proof of concept that IMPDH can be effectively and selectively targeted by small molecule inhibitors. When considering IMPDH from *C. neoformans*, a similar screening approach targeting the co-factor binding site may also be feasible due to its structural and functional deviation from human IMPDH (Morrow et al., 2012). A structure of human IMPDH isoform II bound to selenazole-4-carboxyamide-adenine dinucleotide, an analog of NAD^+^, reveals differences in the residues that comprise the co-factor binding pocket between *C. neoformans* and human IMPDH. It can be inferred that the interaction between IMPDH and NAD^+^ is stabilized by a π-π stacking interaction between the adenine ring of NAD^+^ and R267 and Y296 of *C. neoformans* IMPDH which corresponds to R253 and Y282 in human isoform I or H253 and F282 in human isoform II, respectively (Figure 5A) (Morrow et al., 2012). Although many residues within the binding pocket are invariant between *C. neoformans* and human isoforms I and II, these differences are likely to be sufficient to achieve species-selective inhibition. A high-throughput screen against IMPDH from *C. neoformans* has identified promising candidates (Kummari et al., 2018). From a chemical library of 114,000 drug-like compounds, three 3-((5-substituted)-1,3,4-oxadiazol-2-yl)thio benzo[*b*]thiophene 1,1-dioxides were identified as having inhibitory activity against *C. neoformans* IMPDH. Through chemical modification, several analogs were then synthesized to assess the structure-activity relationships. Although many of these analogs inhibited *C. neoformans* IMPDH *in vitro* and had whole cell activity, they were also cytotoxic to human cell lines (Kummari et al., 2018). These results illustrate the complexities associated with targeting pathways shared between fungi and humans. However, there is also great opportunity to build upon this work by trialing modifications that improve selectivity that retain the antifungal properties of the compound whilst reducing cytotoxic effects.

Similarly to IMPDH, inhibition of fungal GMP synthase may be achieved effectively by high-throughput screening of chemical libraries or fragment screening. Since GMP synthase is a bifunctional protein with two catalytic domains working in tandem to fulfill its function, it is imperative to select the most feasible binding pocket to target. The glutamine amidotransferase (GAT) domain releases ammonia from glutamine which is shuttled to the ATP pyrophosphatase (ATPPase) domain where XMP is converted to GMP *via* an adenyl-XMP intermediate. There are pre-existing inhibitors that target the glutamine amidotransferase domain such as acivicin (ACI) and 6-diazo-5-oxo-L-norleucine (DON) that inadvertently inhibit GMP synthase activity (Ahluwalia et al., 1990; Chittur et al., 2001). However, targeting this catalytic module of GMP synthase would likely lead to non-specific and off-target effects due to the prevalence of GAT domains in other enzymes. Therefore, a more promising endeavor is to study the XMP- and ATP-binding pockets located within the ATPPase domain to achieve potent inhibition and high selectivity for the fungal enzyme over the mammalian equivalent. A novel compound, denoted ECC1385, identified from a synthetic compound library has been shown to inhibit *C. albicans* and *C. neoformans* GMP synthase activity *in vitro* and exhibits whole cell activity against a broad-spectrum of fungi and bacteria, including multiple species of *Candida, A. fumigatus* (MF5668), *C. neoformans* (MY2062), and *Staphylococcus aureus* (MB2865) (Rodriguez-Suarez et al., 2007). Mechanism-of-action studies of ECC1385 have revealed that inhibition of GMP synthase is achieved through a different mode of action from ACI and DON. These data suggest that small chemical compounds that target the activity of the ATPPase domain is possible and presents a promising avenue to pursue.

As NDK has maintained its essential role in NTP biosynthesis throughout all domains of life, there is high structural and functional conservation between different species. This conservation is reflected in the largely invariant composition of binding pocket residues between fungal and human homologs (Figure 5B). However, two key differences are revealed when the crystal structures of ADP-bound NDK from human (2HVD), *A. fumigatus* (6XP7), and *A. flavus* (6K3H) are superimposed (Figure 5B) (Giraud et al., 2006; Nguyen et al., in press). Specifically, in the nucleoside binding pocket, Lys57 and C116 in both fungal NDKs are replaced by Arg58 and I117 in the human structure, respectively. These discrepancies presents an opportunity to selectively target the catalytic site and this may be beneficial in the development of a pan inhibitor with broad-spectrum activity against multiple pathogenic fungi. Azidothymidine (AZT), also marketed as RETROVIR, is an antiretroviral medication currently used to treat HIV (Furman et al., 1986). It has been shown that AZT can inhibit NDK from *A. flavus in vitro* and exhibits antifungal activity *in vivo* (Wang et al., 2019). Docking of AZT to the crystal structure of *A. flavus* NDK predicts that it acts as a competitive inhibitor and interacts with residues R104, H117, and D120 in the catalytic site. Since AZT is an FDA-approved drug, there is potential to repurpose it as an antifungal drug or to use the AZT scaffold and improve upon its antimycotic properties. An alternate approach may be explored to inactivate fungal NDK preferentially over the human homolog using non-competitive inhibitors. Allosteric sites are likely to show more variation between species, compared to the catalytic site, and therefore, development of a non-competitive inhibitor may be a more effective approach. Thorough kinetic analysis has revealed that ebselen, a cysteine-modifying compound, acts as a non-competitive inhibitor of human NDK secreted by airway epithelial cells (Semianrio-Vidal et al., 2010). In this context, it is not used in an antifungal application. Instead, it is used to obtain stable and accurate measurements of nucleotides in airway surface liquid by disrupting nucleotide interconversion without the use of a nucleotide-derived inhibitor. Identifying allosteric sites in NDK from fungal pathogens, comparing them to the human homolog and exploiting their differences may be imperative to simultaneously achieve potent inhibition and high selectivity. Hence, it would be advantageous to explore non-competitive inhibitors of NDK that bind an allosteric site and influence catalytic activity distally.

Although not explicitly addressed in this review, there has been substantial progress made in targeting enzymes from the *de novo* pyrimidine biosynthesis pathway that is worth further discussion. Notably, a novel drug, known as F901318 or Olorofim), has shown antifungal activity against *A. fumigatus* and *A. flavus* by inhibiting the enzyme, dihydroorotate dehydrogenase (Du Pré et al., 2018). Furthermore, it has shown efficacy against *A. fumigatus*, both azole-sensitive and azole-resistant strains, in a murine model of pulmonary aspergillosis (Oliver et al., 2016). F901318 is currently being assessed for its use in treating invasive fungal infections caused by *Lomentospora prolificans, Scedosporium* spp., *Aspergillus* spp., and other resistant fungi. The development of F901318 and progression into phase 2 clinical trials highlights the untapped potential of targeting shared metabolic pathways between fungi and humans for the discovery of novel antifungal drug classes.

## Conclusions

As the number of immunocompromised patients continues to grow, invasive fungal infections are becoming more pervasive. Due to inherent limitations in antifungal drug treatments and the emergence of resistance, the limited spectrum of currently available antifungals is a major barrier to effectively treat systemic fungal infections. Consequently, there is a need to identify and validate novel antifungal drug targets. Targeting eukaryotic proteins that are common to both fungi and humans is a difficult but necessary approach since the number of fungal enzymes that lack a human homolog and are essential for survival or virulence are scarce. Hence, there has been an increased interest in exploiting subtle structural and functional differences of metabolic enzymes shared by both fungi and humans. In the field, significant progress has been made in the characterization of enolase, an enzyme implicated in tissue invasion, as well as enzymes from the mannitol biosynthesis and *de novo* purine biosynthesis pathways in many pathogenic fungi. In addition to the pathways explored in this review, *de novo* pyrimidine biosynthesis enzymes, intracellular redox environment regulators and key phosphatases, kinases and transcription factors that form cellular signaling networks have been explored as potential antifungal drug targets (Oliver et al., 2016; Marshall et al., 2019; Jin et al., 2020). Altogether, these experiments lay the foundation for antifungal drug discovery projects that target unconventional, but promising, pathways.

## Author Contributions

SN, JT, and JB: conceptualization, manuscript review, and editing. SN and JT: manuscript preparation. All authors contributed to the article and approved the submitted version.

## Conflict of Interest

The authors declare that the research was conducted in the absence of any commercial or financial relationships that could be construed as a potential conflict of interest.
